# The Effect of Colloids versus Crystalloids for Goal-Directed Fluid Therapy on Prognosis in Patients Undergoing Noncardiac Surgery: A Meta-Analysis of Randomized Controlled Trials

**DOI:** 10.1155/2024/4386447

**Published:** 2024-06-14

**Authors:** Wang Niu, Junyi Li, Shouping Wang

**Affiliations:** ^1^Department of Anesthesiology, West China Hospital, Sichuan University and The Research Units of West China (2018RU012), Chinese Academy of Medical Sciences, Chengdu, China; ^2^Department of Anesthesia Operating Center, West China Hospital, Sichuan University, West China School of Nursing, Chengdu, China; ^3^Department of Intensive Care Unit, West China Hospital, Sichuan University, Chengdu, China

## Abstract

**Background:**

Goal-directed fluid therapy (GDFT) contributes to improvements in intraoperative fluid infusion based on objective parameters and has been widely recommended in clinical practice. In addition, increasing evidence reveals that GDFT can improve the prognosis of surgical patients. However, considering the individual characteristics of colloids and crystalloids in clinical use, it is uncertain as to which type of fluids administered is associated with better outcomes in the condition of GDFT.

**Objectives:**

To evaluate the effect of colloids versus crystalloids under GDFT on prognosis in patients undergoing noncardiac surgery. *Data Sources*. Randomized controlled trials (RCTs) from PubMed, EMBASE, Ovid MEDLINE, CNKI, Cochrane library, and reference lists of relevant articles.

**Methods:**

Two investigators independently screened and reviewed studies for inclusion and performed data extraction. Our primary outcome was a composite of postoperative complications. The secondary outcomes were (1) mortality at the follow-up duration; (2) postoperative complications of several organ systems, including cardiac, pulmonary, digestive, urinary, nervous system, and postoperative infection events; and (3) hospital and ICU length of stay. Heterogeneity was assessed by the *I*^2^ and chi-square tests. The odds ratio (OR) of the dichotomous data, mean difference (MD) of continuous data, and 95% confidence intervals (CI) were calculated to assess the pooled data.

**Results:**

Of 332 articles retrieved, 15 RCTs (involving 2,956 patients undergoing noncardiac surgery) were included in the final analysis. When the data were pooled, patients in the colloids and crystalloids group revealed no difference in postoperative composite complications (OR = 0.84, 95% CI = 0.51–1.38, *P*=0.49) under GDFT. Regarding the secondary outcomes, patients in the colloids group were associated with fewer digestive system complications (OR = 0.64, 95% CI = 0.41–0.98, *P*=0.04). However, no difference was found in mortality (OR = 1.37, 95% CI = 0.72–2.58, *P*=0.34), complications of the cardiac system (OR = 1.49, 95% CI = 0.66-3.37, *P*=0.34), pulmonary system (OR = 0.89, 95% CI = 0.62–1.28, *P*=0.53), urinary system (OR = 1.05, 95% CI = 0.61-1.80, *P*=0.87), nervous system (OR = 1.04, 95% CI = 0.55–1.98, *P*=0.90), postoperative infection events (OR = 0.89, 95% CI = 0.75–1.07, *P*=0.22), length of hospital stay (difference in mean = −0.71, 95% CI = −1.49–0.07, *P*=0.07), and ICU stay (difference in mean = −0.01, 95% CI = −0.20–0.18, *P*=0.95) between patients receiving GDFT with colloids or crystalloids.

**Conclusion:**

There is no evidence of a benefit in using colloids over crystalloids under GDFT in patients undergoing noncardiac surgery, despite its use resulting in lower digestive system complications.

## 1. Introduction

An important duty of anesthesiologists is to maintain the stability of intraoperative hemodynamics by suitable fluid infusion, which can meet the needs of sufficient organ perfusion. Previous works confirmed that fluid therapy decisions can influence the clinical prognosis of patients [1]. Optimization of perioperative fluid treatment often results in enhanced postoperative outcomes, reduced perioperative complications, and shortened hospitalization [2]. Moreover, fluid administration based on objective indicators of individual responsiveness to liquid therapy can reduce postoperative complications and enhance patient recovery from surgery [3, 4]. This method of fluid administration based on a variety of objective hemodynamic parameters is called goal-directed fluid therapy (GDFT). It operates on flow-based dynamic hemodynamic parameters such as pulse pressure variation (PPV), stroke volume (SV), and stroke volume variation (SVV) [5]. In 1997, Sinclair et al. found that intraoperative intravascular volume loading to optimal SV guided by esophageal Doppler resulted in a more rapid postoperative recovery and a significantly reduced hospital stay [6]. Since then, GDFT has attracted much attention and proved its positive role in various clinical situations [7–9]. However, which type of fluid infusion favors better outcomes in the condition of GDFT is still the subject of debate and remains unclear. In general, two main types of fluid are used intraoperatively in clinical practice: colloids and crystalloids. Crystalloid solutions are the most commonly used fluids because they are inexpensive, readily available, and relatively nontoxic [10]. However, crystalloid solutions have a short half-life and leave the intravascular space within minutes, thus providing little hemodynamic support. It also accumulates in tissues, including the lungs and incision sites, and promotes edema, weight gain, and prolonged recovery, which all worsen the prognosis of surgery patients [10–12]. In contrast, colloids might be more efficient than crystalloids in expanding plasma volume because they are retained within the intravascular space and maintain colloid oncotic pressure longer than crystalloids, thereby reducing tissue edema, but their cost has limited their use [10, 13]. Moreover, colloids may also affect coagulant function and increase the risk of acute kidney injury (AKI) and permeability of the microvasculature under conditions of surgery, inflammation, or trauma. The leakage of colloidal macromolecules into interstitial tissues can also increase interstitial colloid osmotic pressure (which is consistent with its role in the blood vessels), thus exacerbating edema. Previous clinical studies that focused on the efficacy of colloids and crystalloids in the condition of GDFT have yielded different conclusions [14–28] and gained no consensus on the results; thus, we performed a meta-analysis to evaluate the effect of colloids versus crystalloids under GDFT on prognosis in patients undergoing noncardiac surgery.

## 2. Methods

The present meta-analysis adhered to the preferred reporting items for systematic reviews and meta-analysis guidelines [29]. All analyses were based on previously published studies; thus, no ethical approval or patient consent was needed. It has been registered in the international prospective register of systematic reviews (CRD42020153043).

### 2.1. Selection Criteria

The selection criteria are as follows.

#### 2.1.1. Participants

Adult patients (>18 years) undergoing scheduled noncardiac surgery.

#### 2.1.2. Intervention

Intraoperative fluid infusion decision of GDFT with colloids or crystalloids.

#### 2.1.3. Comparison

Patients who received colloids and those who received crystalloids in the condition of GDFT were compared.

#### 2.1.4. Outcomes

The primary outcome was a composite of postoperative complications. The secondary outcomes were (1) mortality at the follow-up duration; (2) postoperative complications of several organ systems, including cardiac, pulmonary, digestive, urinary, nervous, and postoperative infection events; and (3) length of stay at hospital and ICU. Any study containing the primary outcome or secondary outcomes was screened and then included in the final analysis based on its full text.

#### 2.1.5. Study Type

RCT.

### 2.2. Data Sources

RCTs from PubMed, EMBASE, Ovid MEDLINE, CNKI, and Cochrane library were reviewed (last updated in May 2023) to identify eligible studies that met the inclusion criteria by two investigators who searched independently. Moreover, we referred to the references of the included studies for potential trials.

### 2.3. Search Strategy

We used the following search words: “colloids”, “Hydroxyethyl starch”, “crystalloid solutions”, “goal-directed fluid”, “goal-directed fluid therapy”, “goal oriented”, and “goal target” to identify eligible RCTs for inclusion. For example, the detailed search strategy used in PubMed was as follows: (((goal-directed fluid therapy) OR (goal-directed fluid) OR (goal oriented) OR (goal target)) AND ((colloids) OR (Hydroxyethyl Starch))) AND ((Crystalloid Solutions) OR (Ringer's Lactate) OR (Saline Solution)).

### 2.4. Date Extraction

After completing the first step of literature screening to confirm the final data study, two members of the team began to extract data according to the outcome indicators. In order to ensure the accuracy of data extraction, the two members of the data extraction do not know each other's data results. After the data extraction is completed, two members check the data extraction results.

Data abstracted from each individual trial included the population, intervention, comparison, outcome, study type, and other detailed characteristics, such as first author name, year of publication, goal-directed strategy, basal fluid therapy, sample size, and conclusion (Table 1). Our secondary outcomes include postoperative complications of several systems, including cardiac, pulmonary, digestive, urinary, nervous system, and postoperative infection. Among them, complications of the cardiac system include acute heart failure, myocardial infarction, ischemia, arrhythmia, and acute coronary syndrome. Complications of the pulmonary system include pulmonary embolism, pulmonary edema, respiratory failure, pneumonia, pulmonary pleural effusion, and pulmonary effusion. Digestive complications include bowel and surgical anastomosis stricture, anastomotic leak, internal or external fistulas, effusion, gut paralysis, peritonitis, gastrointestinal tract dysfunction, intestinal obstruction, GI failure/small-bowel obstruction, and postoperative nausea and vomiting. Complications of the urinary system include renal dysfunction requiring dialysis, progressive renal insufficiency, and AKI. Complications of the nervous system include transient neurologic disease, stroke, and postoperative confusion. The complications mentioned above and relevant data were all derived from the final included studies.

**Figure 1:**
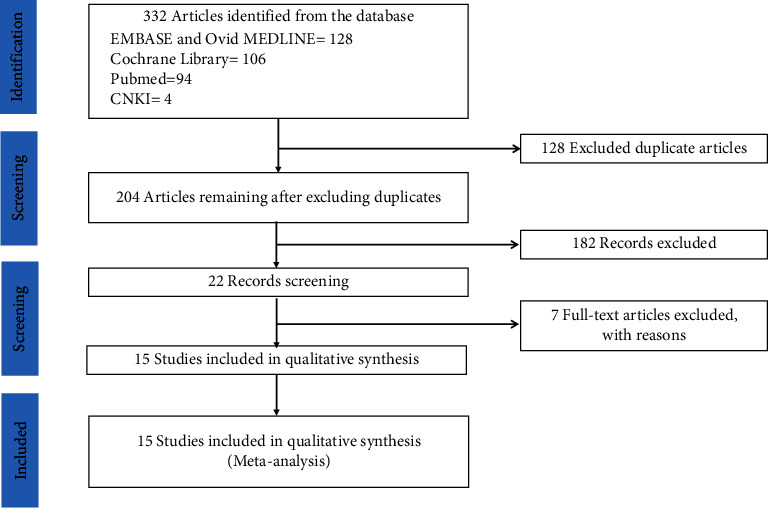
PRISMA-flow diagram for literature search and exclusion criteria.

A total of 332 articles were retrieved by searching PubMed, EMBASE, Ovid MEDLINE, CNKI, the Cochrane library, and reference lists of relevant articles. We excluded 128 duplicate articles and an additional 182 articles that did not meet the selection criteria after reviewing the abstracts. We further excluded seven articles after reviewing the full text. Finally, 15 articles were included in the final analyses.  Figure 1 shows how we identified the required studies according to the PRISMA-flow diagram.

### 2.5. Risk of Bias and Quality of Evidence

**Figure 2:**
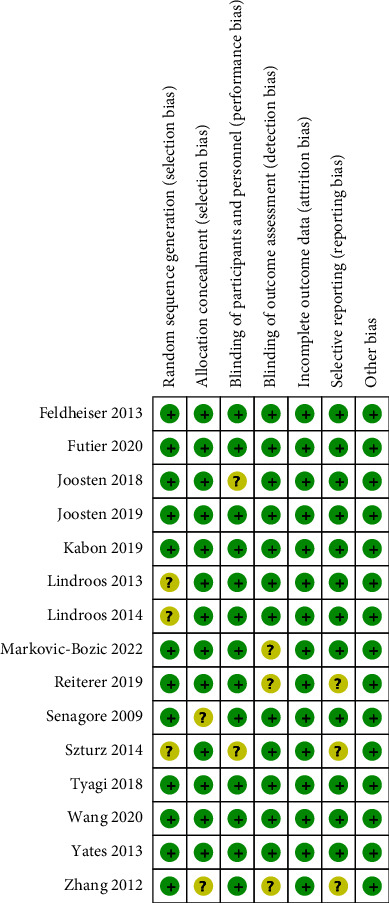
Risk of bias summary.

The assessment of the risk of bias is summarized in Figure 2. Seven studies were judged to be at low risk of bias overall as reviewers judged [16–18, 22, 24, 26, 28], and the remaining eight studies were judged to be at unclear risk of bias. For the key domains, adequate randomized sequences were generated in 12 trials [14–18, 22–28], and appropriate allocation concealment was reported in 13 trials [15–22, 24–28].

**Figure 3:**
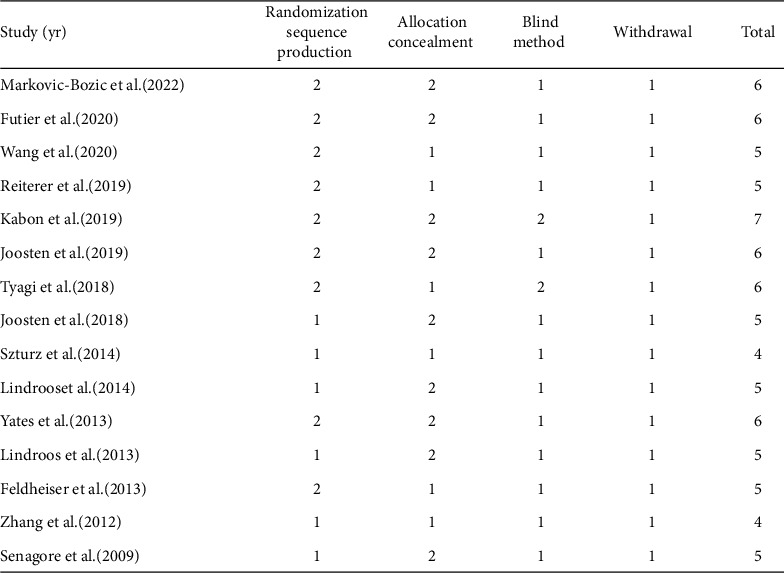
Quality of included studies by Jadad score.

We also assessed the methodological quality of the included studies by the Jadad score, which is considered the optimal valid and reliable tool to assess the methodological quality of a clinical trial [30]. The result of the assessment is shown in Figure 3.

### 2.6. Study Characteristics

The present meta-analysis summarizes the results of RCTs that compare the prognosis of colloid versus crystalloid use in the intraoperative condition of GDFT in noncardiac surgery. In terms of the type of fluids, which were included in the previous studies, all colloids used in the included studies were HES [14–28]. Fifteen RCTs were included in this meta-analysis. The sample size of the included studies ranged between 28 and 1102 and involved 2,956 patients. Among all fifteen trials analysed, ten were performed on abdominal surgery [14–18, 22–25, 28], three were performed on neurosurgery [19, 20, 27], and one study each was performed on urologic [21] and orthopedic surgery [26]. Of them, seven studies described a composite of postoperative complications [15–17, 22–24, 28]. Six report on the nervous system [14, 15, 17, 22, 24, 27]. Seven reported complications of the cardiac [14, 15, 17, 22–24, 28], urinary [14, 15, 17, 22, 24, 26, 28], and digestive [15, 17, 21–25] systems and postoperative infection events [14, 15, 17, 22–24, 28]. Eight reported pulmonary complications [14–16, 22–24, 27, 28]. Ten studies reported data on mortality [14–18, 22–24, 27, 28]. Eleven studies reported the length of hospital or ICU stay [14, 15, 17–20, 22–24, 27, 28]. The information on the outcome indicators contained in each study.

### 2.7. Goal-Directed Fluid Therapy (GDFT) Strategies

All patients in the included study received GDFT by monitoring the stroke volume, LVETi, CI, or PPV. Twelve RCTs maintained SVV < 10% [14–20, 22, 25–28], and one maintained SVV < 13% [24], PPV < 11% [23], and CI between 2.6 and 3.8 L/min/m [21].

### 2.8. Sensitivity Analysis for Primary Outcome

**Figure 4:**
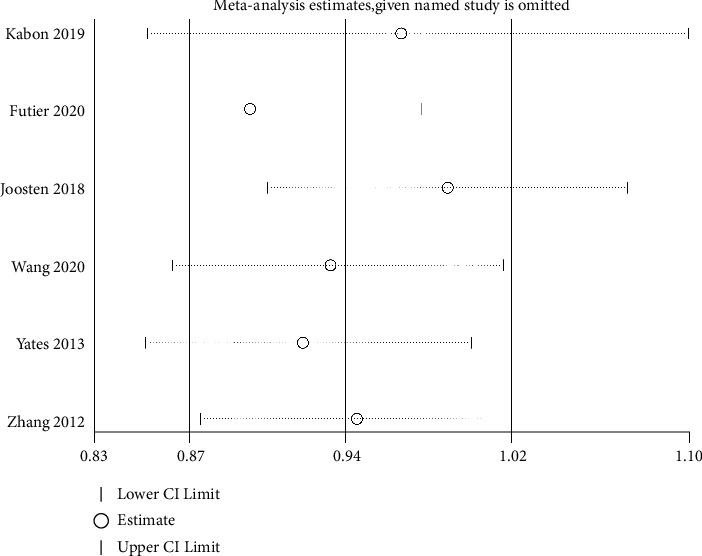
Sensitivity analysis for primary outcome.

STATA16.0 software was used for performing the sensitivity analysis for the primary outcome and the result is presented as Figure 4. The results of four studies were stable, and the results of two studies were slightly unstable. Overall, the results are relatively reliable.

### 2.9. Statistical Analysis

We used review manager (RevMan for Windows, version 5.3; Cochrane Collaboration, Oxford, UK) to perform most analyses. We calculated the odds ratio (OR) for dichotomous (postoperative complications and mortality) and mean differences (MDs) with 95% CIs for continuous (hospital and ICU length of stay) data. We quantified statistical heterogeneity using the *I*^2^ and chi-square statistics and considered heterogeneity to be substantial if the *I*^2^ value was greater than 50%. The results of the study are presented in the form of a random-effects model. We considered a *P* value < 0.05 to be statistically significant. If the median and interquartile range (IQR) were reported in a study, we assumed that the median of the outcome variable was equal to the mean response and that the interquartile range was approximately 1.35 standard deviations in width [31]. We also performed a sensitivity analysis for the primary outcome using STATA16.0 software.

## 3. Results

### 3.1. Primary Outcome

**Figure 5:**
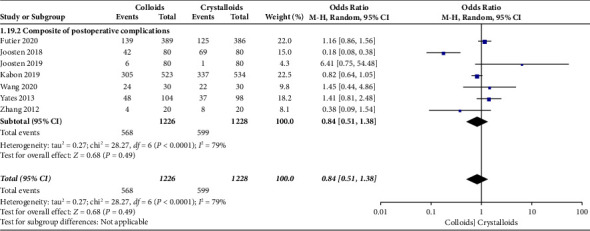
A composite of postoperative complications.

Our primary outcome was a composite of postoperative complications. When the data were pooled, seven studies [15–17, 22–24, 28] evaluated the effect of colloids or crystalloids on the composite of postoperative complications. The results revealed that GDFT with colloids provided no benefits over crystalloids in terms of the composite of postoperative complications (OR = 0.84, 95% CI = 0.51–1.38, *P*=0.49), as presented in Figure 5.

### 3.2. Secondary Outcomes

The secondary outcomes were (1) mortality at the follow-up duration; (2) postoperative complications of several organ systems, including cardiac, pulmonary, digestive, urinary, nervous system, and postoperative infection events; and (3) length of stay at hospital and ICU. The results are presented in Figures 6–14.

### 3.3. Mortality

**Figure 6:**
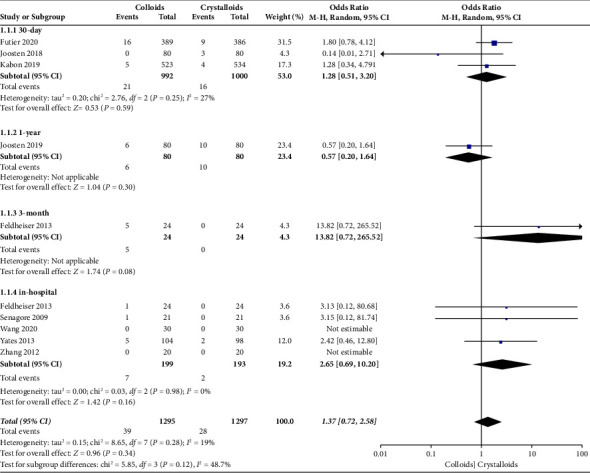
Mortality in the follow-up period.

When the data were pooled, ten studies [14–18, 22–24, 27, 28] evaluated the effect of colloids or crystalloids on mortality in patients with GDFT. The results are presented in Figure 6, and no difference was found (OR = 1.37, 95% CI = 0.72-2.58, *P*=0.34). Specifically, our subgroup analysis included four RCTs [15, 17, 27, 28] that evaluated 30-day mortality (OR = 1.28, 95% CI = 0.51–3.20, *I*^2^ = 27%, *P*=0.59) and five RCTs that evaluated in-hospital mortality (OR = 2.65, 95% CI = 0.69–10.20, *I*^2^ = 0%; *P*=0.16).

### 3.4. Cardiac Complications

**Figure 7:**
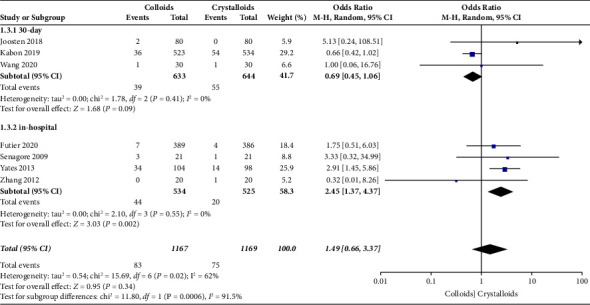
Cardiac system complications.

In our pooled data, seven RCTs [14, 15, 17, 22–24, 28] included cardiac complications, which consisted of acute heart failure, myocardial infarction, ischemia, arrhythmia, and acute coronary syndrome, as presented in Figure 7. The results revealed no difference in cardiac complications in patients receiving GDFT with colloid or crystalloid (OR = 1.49, 95% CI = 0.66–3.37, *I*^2^ = 62%, *P*=0.34). Further subgroup analysis revealed a significant difference in cardiac complications during hospitalization (OR = 2.45, 95% CI = 1.37–4.37, *I*^2^ = 0%; *P*=0.002) between the colloid and crystalloid groups [14, 22, 23, 28]. However, subgroup analysis indicated no significant difference in cardiac complications between the colloid and crystalloid groups 30 days after surgery (OR = 0.69, 95% CI = 0.45–1.06, *I*^2^ = 0, *P*=0.09) [15, 17, 24].

### 3.5. Pulmonary Complications

**Figure 8:**
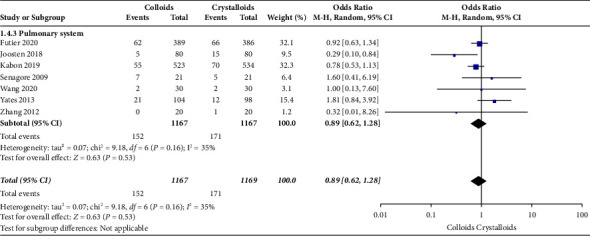
Pulmonary system complications.

Eight RCTs [14, 15, 17, 22–24, 27, 28] evaluated pulmonary system complications after receiving colloids or crystalloids to achieve GDFT and observed no significant difference (OR = 0.89, 95% CI = 0.62–1.28, *P*=0.53). The result is presented in Figure 8.

### 3.6. Digestive Complications

**Figure 9:**
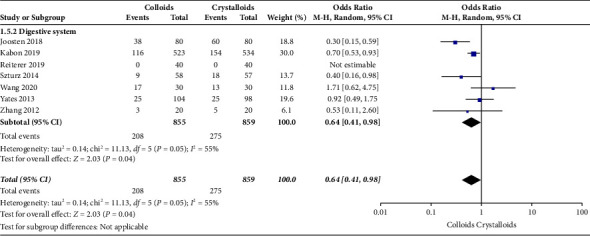
Digestive system complications.

Seven RCTs [15, 17, 21–25] evaluated the frequency of digestive system complications after receiving GDFT with colloids or crystalloids. The results indicated fewer digestive system complications with colloids under GDFT than with crystalloids (OR = 0.64, 95% CI = 0.41–0.98, *P*=0.04), as presented in Figure 9.

### 3.7. Urinary Complications

**Figure 10:**
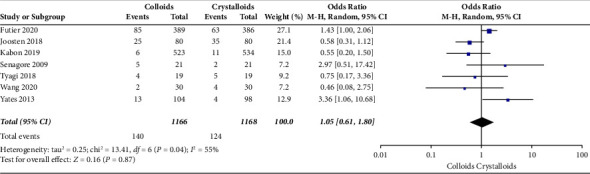
Urinary system complications.

Seven RCTs [14, 15, 17, 22, 24, 27, 28] evaluated urinary system complications after receiving GDFT with colloids or crystalloids and observed no significant difference (OR = 1.05, 95% CI = 0.61–1.80, *P*=0.87), which is presented in Figure 10.

### 3.8. Nervous System Complications

**Figure 11:**
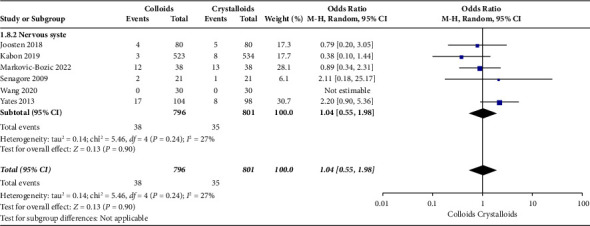
Nervous system complications.

Figure 11 shows the results of nervous system complications assessed by six RCTs [14, 15, 17, 22, 24, 27]. We observed no significant difference concerning infusing colloid or crystalloid under GDFT (OR = 1.04, 95% CI = 0.55-1.98, *P*=0.90).

### 3.9. Postoperative Infection Events

**Figure 12:**
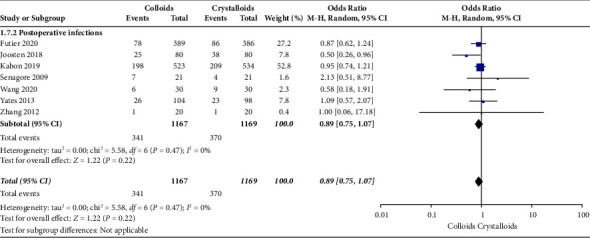
Postoperative infection events.

Postoperative infection events were assessed by seven RCTs [14, 15, 17, 22–24, 28]. As indicated in Figure 12, our pooled data observed no significant difference concerning infusing colloid or crystalloid under GDFT (OR = 0.89, 95% CI = 0.75–1.07, *P*=0.22).

### 3.10. Length of Hospital or ICU Stay

**Figure 13:**
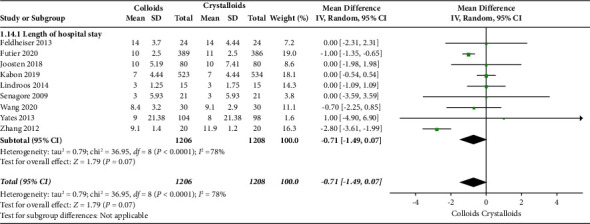
Length of hospital stay.

**Figure 14:**
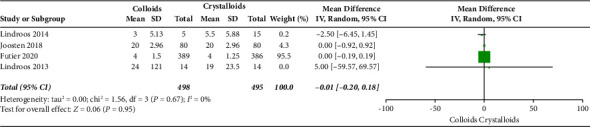
Length of ICU stay.

The length of hospital stay was assessed in ten RCTs [14, 15, 17–19, 22–24, 27, 28]. Pooled analysis revealed a significant difference after receiving GDFT with colloids compared with crystalloids (difference in mean = −0.71, 95% CI = −1.49-0.07, *P*=0.07), as shown in Figure 13. Four RCTs [15, 19, 20, 28] assessed the length of ICU stay and found no significant difference in the colloid and crystalloid groups under the condition of GDFT (difference in mean = −0.01, 95% CI = −0.20-0.18, *P*=0.95), as shown in Figure 14.

## 4. Discussion

In the present meta-analysis, we included 15 RCTs involving 2,956 patients undergoing noncardiac surgery under the condition of GDFT with colloids or crystalloids. The results indicated that GDFT with colloids reduces digestive system complications compared with crystalloids. However, in terms of the composite of, mortality and postoperative complications, including cardiac system, pulmonary system, urinary system, nervous system, and postoperative infection events, and length of hospital or ICU stay, colloids under the GDFT protocol do not offer any additional benefits over crystalloids in this population.

Adequate fluid management during surgery is of utmost importance to maintain adequate perfusion and oxygen delivery to tissues. Traditional fluid therapy protocols based on static hemodynamic targets such as central venous pressure and delayed volume status indexes, such as blood pressure, heart rate, and urine output, often lack definitive physiologic targets for determining optimal fluid decisions. In contrast, GDFT relied on sophisticated dynamic measures of volume status and may serve as a better choice for perioperative fluid administration. Horgan et al. [32] reported a shorter hospital stay and decreased morbidity in patients undergoing elective colorectal resection with a protocol-based fluid optimization programme using intraoperative oesophageal Doppler monitoring. In a well-conducted meta-analysis of eight RCTs, Javier et al. [33] reported a significant reduction in mortality associated with GDFT compared with conventional fluid therapy in adult noncardiac surgery patients. Another meta-analysis of 76 trials [34] suggested that GDFT during general anesthesia might decrease mortality, length of stay at hospital, and several postoperative complications. However, there is no consensus regarding which type of fluid is associated with better outcomes among patients who undergo GDFT. Colloids and crystalloids all have their own advantages and disadvantages; for example, colloids have longer intravascular persistence and volume expanding effects, thus resulting in lower volume requirements and less extravascular edema but causing more anaphylaxis and adverse renal and coagulation effects [35]. In contrast, crystalloid solutions are much cheaper, conveniently acquired, and relatively nontoxic but provide little hemodynamic support and promote edema and weight gain, thus prolonging recovery.

In 2015, Ripollés et al. published a meta-analysis [36] that included six RCTs concerning the effect of colloids versus crystalloids for GDFT on prognosis in patients undergoing noncardiac surgery and observed no positive conclusions. However, this research failed to explore the detailed complications of individual systems, and the data pooled in this meta-analysis are extremely limited (only two studies included information about relevant outcomes). Thus, we performed the present meta-analysis to explore the detailed prognosis, which included a composite of postoperative complications, mortality, complications of individual systems, and length of hospital/ICU stay, by comparing the effects of colloid and crystalloid administration in patients undergoing noncardiac surgery for GDFT.

The findings of our meta-analysis suggest that patients who received colloids to achieve GDFT were associated with fewer digestive complications (*P*=0.04). The reason why colloid infusion is associated with fewer digestive complications may be because intraoperative GDFT with accurate targeting of fluid may prevent excessive fluid administration. Research shows that 16% of colloids and more than 68% of the saline solution escaped into the extravascular fluid compartment 1 h after the infusion [37]. However, large amounts of infused fluids were less effective and impaired gastrointestinal function. Edema of the intestines and other tissues may be responsible for poor tissue oxygenation and postoperative gut dysfunction, thus contributing to increased digestive system complications. The present meta-analysis found a lower length of hospital stay in the colloid group. Zhang et al. [23] also reported that GDFT with colloids decreased the length of hospital stay compared with crystalloids because their study proved a lower number of postoperative complications in the colloid group. However, this meta-analysis found a similar risk of postoperative complications between the two groups and a high heterogeneity (*I*^2^ = 76); thus, this result must be carefully interpreted as a negative result.

It is worth noting that although the present meta-analysis revealed no significant difference in complications of the cardiac system, the results of the subgroup analysis indicated that colloids under GDFT were associated with a higher risk frequency of cardiac complications (*P*=0.002) with no heterogeneity (*I*^2^ = 0) during the in-hospital period. When our team combed through the data from each study, we found that the results were probably influenced by Yates et al.'s findings: in this study, 34 adverse cardiac events occurred in the colloid group, while only 14 occurred in the crystalloid group, far exceeding the incidence seen in other studies. Unfortunately, the Yates's study does not analyze and explain the reason of differences in the cardiac system complications. Considering large molecular weight and difficulty crossing the endothelium of colloids, solutions are expected to remain longer in the intravascular space by using colloids than crystalloids [38]. For example, McIlroy and Kharasch [37] found colloids were associated with an intravascular volume expansion effect twice as large as that of the crystalloid solution. What's more, results from a subanalysis of a large multicenter randomized trial by Kabon et al. [39] revealed that the cardiac index increased significantly more immediately after a colloid bolus administration as compared to a crystalloid bolus administration. However, their results suggested that the time-weighted average in the cardiac index was only 200 mL higher in the colloids group as compared to the crystalloids group which means a statistically significant but clinically unimportant difference in the flow-driven parameters was found. In this context, the net effect of intraoperative goal-directed hydroxyethyl starch administration on the cardiac index is too small to have a relevant impact on clinical outcome, such as complications of the cardiac system. Also, in the trial conducted by Feldheiser [18], the beneficial intraoperative effect by the HES colloid solution included a higher cardiac output compared with crystalloids. However, there were no signs of clinical benefit in terms of a reduction in postoperative complications by using a goal-directed hemodynamic algorithm to optimize stroke volume with balanced starch compared with balanced crystalloid. Therefore, it seemed reasonable that the administration of fluids under GDFT protocol might be more important, whereas the type of fluid administered during surgery plays a much smaller role than that was initially assumed.

Although the intensive care setting reported potential nephrotoxic effects and kidney injury in critical and sepsis patients with colloids [40–42], studies performed in a surgical context have not been reported, and the specific effect of colloids on kidney function is still uncertain. In this meta-analysis, we extracted information about urinary system complications in seven RCTs [14, 15, 17, 22, 24, 26, 28] of 15 included trials and observed no significant difference between the colloid and crystalloid groups under GDFT. It is worth mentioning that our included trials reported a low rate of AKI, and the situation of GDFT is not a critical and septic population; thus, our results indicated that more research is needed to explore the effects of colloid infusion on long-term kidney function in surgical conditions. Previous publications have reported that providing between 1.75 and 2.75 L of crystalloid fluid during open abdominal surgery will not increase the total number of postoperative complications [43]. However, increasing the crystalloid fluid load to 6-7 L in colon surgery increases the risk of pulmonary complications [44]. Theoretically, a large amount of crystalloids is usually needed to achieve hemodynamic stability to meet GDFT. Considering that the adverse effects of crystalloid fluids are usually related to their preferential distribution to specific interstitial areas, such as the lungs, we explored the difference in pulmonary complications between the colloid and crystalloid groups and found no significant difference. The reason for this result may be that the studies included in this meta-analysis did not use such a large amount of total liquid.

Finally, it is important to note that the benefits of the GDFT strategy have been demonstrated by numerous clinical studies, and relevant societies have recommended the GDFT strategy during surgery. Therefore, in the case of perioperative GDFT implementation, patients will theoretically not present hypovolemia, which means that the differences that will be caused by the total amount of fluid input and the type of fluid selected under the premise of GDFT will not be sufficient to produce clinically significant statistical differences.

## 5. Limitation

Several limitations of this meta-analysis should be mentioned. First, current evidence lacks RCTs of high quality, and the sample sizes of the included studies are generally small; thus, the pooled results potentially lack credibility. Secondly, the secondary outcomes of this meta-analysis were postoperative complications, which were calculated as the frequency of complications occurring in each included study, and this counting method may resulting double counting. For example, if a patient experiences pulmonary edema and respiratory complications at the same time, we will include them as postoperative pulmonary complications twice. In addition, the complications reported in various studies were possibly explored using a different definition, and the possibility of inaccurate data could not be ruled out. Last but not least, the clinical use of HES will be greatly limited due to the range of adverse reactions that HES are prone to, such as renal impairment, allergic reactions, and coagulation dysfunction. Thus, given the aforementioned limitations, the conclusion of this meta-analysis should be considered carefully.

## 6. Conclusion

There is no evidence of a benefit in using colloids over crystalloids under GDFT in patients undergoing noncardiac surgery, despite their use resulting in lower digestive system complications.

## Figures and Tables

**Table 1:** Basic information of each individual trial.

Author/Year	Study design	Goal-directed strategy	Basal fluid therapy	Participants	Intervention	Compare	Primary outcomes	Conclusion
Markovic-bozic et al. [27] (2022)	RCT	EVA 1000/FloTrac system guided (additional boluses of 250 mL fluid were given when SVV measured by EVA 1000/FloTrac system rose above 10%)	2 to 4 mL/kg/h of balanced crystalloid regimes	Adult patients that underwent brain tumour surgery	GDFT through 1000/FloTrac system based on algorithm of SVV with HES *n* = 38	GDFT through 1000/FloTrac system based on algorithm of SVV with balanced crystalloid *n* = 38	The impact of type and consumption of fluid on the incidence of perioperative complications	If protocol perioperative haemodynamic management is used, the type of fluid does not have significant impact on the outcome
Futier et al. [28] (2020)	RCT	Individualized goal-directed therapy protocol guided (if the SV increased >10%, the bolus of 250 mL HES or saline was administered to maintain SVV < 10%)	4 mL/kg/h as maintenance fluid during surgery	Adult patients aged 18 years or older admitted for elective or nonelective abdominal surgery	GDFT through individualized goal-directed therapy protocol with HES *n* = 389	GDFT through individualized goal-directed therapy protocol with saline *n* = 386	A composite of death or preselected major postoperative complications	Among patients at risk of postoperative kidney injury undergoing major abdominal surgery, use of HES for volume replacement therapy compared with 0.9% saline resulted in no significant difference in a composite outcome of death or major postoperative complications within 14 days after surgery
Wang [24] (2020)	RCT	Flo Trac/Vigileo system guided (additional boluses of 200 mL fluid were given when SVV > 13 measured by flo Trac/Vigileo system)	4 mL/kg/h of 0.9% saline for maintenance	Patients who underwent elective da Vinci robot-assisted abdominal surgery	GDFT through flo Trac/Vigileo system based on algorithm of SVV with HES *n* = 30	GDFT through flo Trac/Vigileo system based on algorithm of SVV with lactated Ringer's *n* = 30	Postoperative morbidity survey (POMS)	When GDFT was used in patients undergoing robotic abdominal surgery, the incidence of postoperative complications is similar between crystalloid and colloid
Reiterer et al. [25] (2019)	RCT	Doppler-guided (if the SV increased >10%, the bolus of 200 mL SF was administered to maintain SVV < 10%)	5 to 7 mL/kg of lactated Ringer's solution during anesthetic induction, followed by 3 to 5 mL/kg/h for maintenance	Patients (age 18 to 80 years) scheduled for elective moderate- to high-risk open abdominal surgery	GDFT through CardioQ® based on algorithm of SV with HES *n* = 40	GDFT through CardioQ® based on algorithm of SV with lactated Ringer's *n* = 40	Postoperative Psqo2 measured in the surgical wound	Goal-directed colloid administration did not increase Psqo2 compared with goal-directed crystalloid administration in patients undergoing open abdominal surgery
Kabon et al. [17] (2019)	RCT	Doppler-guided (if the SV increased >10%, the bolus of 250 mL SF was administered to maintain SVV < 10%)	5 to 7 mL/kg of lactated Ringer's solution during anesthetic induction, followed by 3 to 5 mL/kg/h for maintenance	Patients (age 18 to 80 years) undergoing abdominal surgery	GDFT through CardioQ® based on algorithm of SV with HES *n* = 549	GDFT through CardioQ® based on algorithm of SV with lactated Ringer's solution *n* = 553	Composite of serious complications	Doppler-guided intraoperative HES administration did not significantly reduce a composite of serious complications
Joosten et al. [16] (2019)	RCT	Closed-loop software guided (if the SV increased >15%, the bolus of 100 mL was administered to maintain SVV < 10%)	3 mL/kg/h of isotonic balanced crystalloid for maintenance	Adult patients undergoing elective major open abdominal surgery	GDFT through closed-loop software based on algorithm of SV with HES *n* = 80	GDFT through closed-loop software based on algorithm of SV with crystalloid solution *n* = 80	Renal function and WHODAS score	There was no evidence of a statistically significant difference in long-term renal function between a balanced hydroxyethyl starch and a balanced crystalloid solution
Tyagi et al. [26] (2018)	RCT	Vigileo^TM^ system guided (100 mL of 6% HES or Ringer's lactate boluses when SVV >10% in supine or lateral position, or >14% in prone position)	2 mL/kg/h of Ringer's solution for maintenance	Adult patients (age 18 to 65 years) scheduled for major orthopedic surgery under general anesthesia	GDFT through VigileoTM system based on algorithm of SVV with HES *n* = 19	GDFT through VigileoTM system based on algorithm of SVV with Ringer's lactate *n* = 19	Incidence of early postoperative AKI	SVV‐guided tetrastarch and Ringer's lactate do not result in postoperative AKI diagnosed by urinary NGAL >100 ng/mL; however, an insignificant trend for better renal functions as well as significantly more efficacious volume expansion and hemodynamic stability were seen with tetrastarch instead
Joosten et al. [15] (2018)	RCT	Closed-loop software guided (if the SV increased >15%, the bolus of 100 mL SF was administered to maintain SVV < 10%)	3 mL/kg/h of isotonic balanced crystalloid for maintenance	Adult patients undergoing elective major open abdominal surgery	GDFT through closed-loop software based on algorithm of SV with HES *n* = 80	GDFT through closed-loop software based on algorithm of SV with crystalloid solution *n* = 80	The postoperative morbidity survey score	A colloid-based goal-directed fluid therapy was associated with fewer postoperative complications than a crystalloid one
Szturz et al. [21] (2014)	RCT	Doppler-guided (if LVETi <400 ms, the bolus of 250/500 mL was administered to maintain CI between 2.6 and 3.8 L/min/m^2^)	No mention	Patients (age >21 years) undergoing major elective urological surgery	GDFT based on Hemosonic^TM^ 100® according to TED variables with HES *n* = 58	GDFT based on Hemosonic^TM^ 100® according to TED variables with Ringer's solution *n* = 57	The comparison of volume replacement efficiency	The significant difference between volumes of crystalloids and colloids proved their different characteristics such as unequal distribution between compartments
Lindroos et al. [20] (2014)	RCT	SV guided (if the stroke volume increased >10%, the bolus of 100 mL SF was administered to maintain SVV < 10%)	3 mL/kg/h of lactated Ringer's solution before anesthetic induction, followed by 200 mL. 1 mL/kg/h of lactated Ringer's solution until the following morning	Adult patients undergoing elective neurosurgery in the prone position	GDFT through Flotrac ® based on algorithm of maximization of SV with HES; *n* = 15	GDFT through Flotrac ® based on algorithm of maximization of SV with Ringer acetate *n* = 15	The volumes required for stable hemodynamics and possible coagulatory effects	We suggest cautious administration of HES during neurosurgery
Yates et al. [22] (2013)	RCT	LiDCO rapid monitor (if the stroke volume increased >10%, the bolus of 250 mL SF was administered to maintain SVV < 10%)	1.5 mL/kg/h of Hartmann's solution from the start and continued for 24 h	Patients (age >55 years) undergoing elective colorectal surgery	GDFT through LiDCO rapid ® based on algorithm of maximization of SVV with HES *n* = 104	GDFT through LiDCO rapid ® based on algorithm of maximization of SVV with Ringer Lactate *n* = 98	Presence of GI morbidity	There is no evidence of a benefit in using HES over crystalloid
Lindroos et al. [19] (2013)	RCT	SV guided (if the stroke volume increased >10%, the bolus of 100 mL SF was administered to maintain SVV < 10%)	3 mL/kg/h of lactated Ringer's solution before anesthetic induction, followed by 200 mL. 1 mL/kg/h of lactated Ringer's solution until the following morning	Adult patients undergoing elective craniotomy in the sitting position	GDFT through Flotrac ® based on algorithm of maximization of SV with HES *n* = 14	GDFT through Flotrac ® based on algorithm of maximization of SV with Ringer Acetate *n* = 14	The volumes required for stable haemodynamics and possible effects on the coagulation	Fluid filling with HES boluses resulted in a positive response in CI and SVI during the sitting position
Feldheiser et al. [18] (2013)	RCT	Doppler guided (if the SVV >10%, the bolus of 200 mL SF was administered to maintain SVV < 10%)	3 to 5 mL/kg of balanced crystalloid during anesthetic induction	Adult ovarian cancer patients undergoing cytoreductive surgey	GDFT through CardioQ® based on maximization algorithm of SV with balanced HES *n* = 24	GDFT through CardioQ ® based on maximization algorithm of SV with balanced crystalloids (Jonosteril ®) *n* = 24	The amount of i.v. administered study fluids used during surgery	Using a goal-directed haemodynamic algorithm to optimize stroke volume, a balanced HES solution is associated with better haemodynamic stability and reduced need for fresh-frozen plasma. There were no signs of renal impairment by colloid solutions when fluid administration is targeted to optimize cardiac preload
Zhang et al. [23] (2012)	RCT	PPV guided (if the PPV was >11%, the bolus of 250 mL SF was administered to maintain SVV < 10%)	4 mL/kg/h of lactated Ringer's solution was infused intraoperative. 1.5–2 mL/kg/h of lactated Ringer's solution until the postoperative day 3	Patients (age 18 to 64 years) undergoing elective gastrointestinal surgeries	GDFT based on optimization PPV <10% with HE *n* = 20	GDFT based on optimization of PPV <10% with Ringer lactate *n* = 20	Length of hospital stay	Monitoring and minimizing pulse pressure variation by 6% hydroxyethyl starch solution (130/0.4) loading during gastrointestinal surgery improves postoperative outcomes and decreases the discharge time of patients
Senagore et al. [14] (2009)	RCT	Doppler guided (if the SVV >10%, the bolus of 200 or 300 mL SF was administered to maintain SVV < 10%)	5 mL/kg of lactated Ringer's solution before anesthetic induction, followed by 5 mL/kg/h of lactated Ringer's solution for maintenance	Patients(age 18 to 80 years) undergoing elective laparoscopic segmental colectomy	GDFT through CardioQ ® based on algorithm of maximization of SV with HES *n* = 21	GDFT through CardioQ ® based on algorithm of maximization of SV with Ringer lactate *n* = 21	Length of hospital stay	Goal-directed fluid management with a colloid/balanced salt solution offers no advantage and is more costly

## Data Availability

The data used to support the findings of this study are available from the corresponding author upon reasonable request.

## Abbreviations

GDFT: Goal-directed fluid therapy
RCT: Randomized controlled trial
MD: Mean difference
OR: Odds ratio
CI: Confidence intervals
PPV: Pulse pressure variation
SV: Stroke volume
SVV: Stroke volume variation
AKI: Acute kidney injury
GI: Gastrointestinal
PRISMA: Preferred reporting items for systematic reviews and meta-analysis
ICU: Intensive care unit
LVETi: Left ventricular ejection time index
CI: Cardiac index
SF: Study fluids
CNKI: China National Knowledge Infrastructure.
